# Shiga Toxin Glycosphingolipid Receptors in Human Caco-2 and HCT-8 Colon Epithelial Cell Lines

**DOI:** 10.3390/toxins9110338

**Published:** 2017-10-25

**Authors:** Ivan U. Kouzel, Gottfried Pohlentz, Julia S. Schmitz, Daniel Steil, Hans-Ulrich Humpf, Helge Karch, Johannes Müthing

**Affiliations:** 1 Institute for Hygiene, University of Münster, D-48149 Münster, Germany; ivan.kouzel@uni-muenster.de (I.U.K.); pohlentz@uni-muenster.de (G.P.); Daniel.Steil@ukmuenster.de (D.S.); Helge.Karch@ukmuenster.de (H.K.); 2 Interdisciplinary Center for Clinical Research (IZKF), University of Münster, D-48149 Münster, Germany; 3 Institute of Food Chemistry, University of Münster, D-48149 Münster, Germany; SchmitzJulia@gmx.de (J.S.S.); humpf@uni-muenster.de (H.-U.H.)

**Keywords:** Caco-2, colon epithelial cells, EHEC, glycolipids, HCT-8, receptor, Shiga toxin, Stx2a, Vero-B4 cells

## Abstract

Shiga toxins (Stxs) released by enterohemorrhagic *Escherichia coli* (EHEC) into the human colon are the causative agents for fatal outcome of EHEC infections. Colon epithelial Caco-2 and HCT-8 cells are widely used for investigating Stx-mediated intestinal cytotoxicity. Only limited data are available regarding precise structures of their Stx receptor glycosphingolipids (GSLs) globotriaosylceramide (Gb3Cer) and globotetraosylceramide (Gb4Cer), and *lipid raft* association. In this study we identified Gb3Cer and Gb4Cer lipoforms of serum-free cultivated Caco-2 and HCT-8 cells, chiefly harboring ceramide moieties composed of sphingosine (d18:1) and C16:0, C22:0 or C24:0/C24:1 fatty acid. The most significant difference between the two cell lines was the prevalence of Gb3Cer with C16 fatty acid in HCT-8 and Gb4Cer with C22–C24 fatty acids in Caco-2 cells. Lipid compositional analysis of detergent-resistant membranes (DRMs), which were used as *lipid raft*-equivalents, indicated slightly higher relative content of Stx receptor Gb3Cer in DRMs of HCT-8 cells when compared to Caco-2 cells. Cytotoxicity assays revealed substantial sensitivity towards Stx2a for both cell lines, evidencing little higher susceptibility of Caco-2 cells versus HCT-8 cells. Collectively, Caco-2 and HCT-8 cells express a plethora of different receptor lipoforms and are susceptible towards Stx2a exhibiting somewhat lower sensitivity when compared to Vero cells.

## 1. Introduction

Shiga toxins (Stxs) released by Stx-producing *Escherichia coli* (STEC) are primary virulence factors in the pathogenesis of hemorrhagic colitis and potentially fatal extraintestinal complications such as hemolytic uremic syndrome and central nervous system sequelae [1,2,3,4,5,6]. Stxs are multifunctional toxins with AB_5_ structure [7], which play an important biological role in microbe defense against protist predators such as *Tetrahymena thermophila* and *Arcanthamoeba castellanii* [8,9,10] suggesting that mammals are not the primary targets of Stxs. However, during human STEC infections, Stxs are released into the gut, enter the bloodstream and target the renal endothelium [11,12,13]. There is no consensus on the mechanism by which Stx reach the endothelia of the target organs, although the functional role of polymorphonuclear leukocytes as Stx carrier in the circulation has been indicated [14,15,16]. A method has been described for detection of the functional activity of Stx in sera of STEC-infected patients during hemorrhagic colitis [17]. This approach could be useful for studying the presence of Stx in different blood fractions such as neutrophils, monocytes, platelets, and leukocyte-platelet aggregates as well as microvesicles and/or lipoproteins [16,18,19,20,21,22,23,24,25,26,27] indicating the multifaceted mechanisms and vehicles by which Stx may be distributed through the human body.

The so far described Stxs of type 1 with 3 subtypes (Stx1a, Stx1c and Stx1d) and of type 2 with seven subtypes (Stx2a–Stx2g) (for appropriate nomenclature of the various Stx subtypes, refer to Scheutz et al., 2012 [28]) consist of a ~32 kDa A-subunit non-covalently linked to a pentamer of five identical ~7.7 kDa sized B-subunits [4,29], which function as a delivery tool for the cytotoxic A-moiety to intracellular target structures. All Stxs analyzed to date preferentially bind to the glycosphingolipid (GSL) globotriaosylceramide (Gb3Cer, Galα1-4Galβ1-4Glcβ1-1Cer) and to a more or less extent to the low-affinity receptor globotetraosylceramide (Gb4Cer, GalNAcβ1-3Galα1-4Galβ1-4Glcβ1-1Cer) [30] with the exception of subtype Stx2e, which prefers Gb4Cer as the major receptor GSL [31] and exhibits promiscuous binding towards extended globo-series GSLs such as the Forssman GSL (GalNAcα1-3GalNAcβ1-3Galα1-4Galβ1-4Glcβ1-1Cer) [30] and globopentaosylceramide (Gb5Cer, Galβ1-3GalNAcβ1-3Galα1-4Galβ1-4Glcβ1-1Cer) [32]. Upon binding to the plasma membrane, Stx is internalized by both clathrin- and dynamin-dependent and independent pathways, transported by a retrograde pathway via the early endosome through the Golgi apparatus to the endoplasmic reticulum and translocated to the cytosol, where the enzymatically active moiety exerts its toxic function [7,33,34,35,36,37]. The cytotoxic action of Stxs rests upon their *N*-glycosidase activity that depurinates not only a specific adenine in a conserved loop of the large rRNA and results in the inhibition of protein biosynthesis, but shows also depurination activity towards nuclear DNA [38] and acts as a DNA repair inhibitor [39,40]. Furthermore, Stxs are also capable of activating multiple cell stress signaling pathways [41].

Human microvascular endothelial cells that line the small blood vessels are canonical target cells in the kidney and other organs due to the expression of GSLs of the globo-series [42,43,44,45]. The clinically most relevant Stx subtypes 1a and 2a preferentially bind to Gb3Cer and to less extent to Gb4Cer [30]. The Stx receptor GSLs Gb3Cer and Gb4Cer have been analyzed in detail in the past by us and others in endothelial cell lines and primary endothelial cells originating from different vascular beds [46,47,48,49,50,51,52,53,54,55] and, more recently, in primary human brain and kidney endothelial cells providing novel details on the fine structure of Stx receptors and their association with *lipid rafts* [56,57].

The presence of Stx GSL receptors in epithelial cells of the human gut and their possible functional role during infections of enterohemorrhagic *E. coli* (EHEC), the human–pathogenic subgroup of STEC, is controversially discussed and still a matter of debate [58]. Human intestinal epithelium represents the first point of contact of released Stx with the host and furthermore acts as a barrier by preventing toxin access to the systemic circulation. Normal human small and large intestinal epithelial cells have been found being negative for the expression of Gb3Cer or any other Stx receptors [59,60,61]. In contrast, binding of Stx1a and Stx2a (formerly named Stx1 and Stx2) to Gb3Cer and Gb4Cer has been detected in human colonic epithelia in fresh tissue sections suggesting the presence of small quantities of Gb3Cer in human colonic epithelia, where it may compete for Stx binding with the more abundantly expressed Gb4Cer [62]. Furthermore, overexpression of Gb3Cer has been found to be associated with malignancy and metastasis of the human colon epithelium [63,64,65,66]. Consequently, the possible use of Stx for therapy of colon cancer [5,7,35,67] and other tumor entities [68,69,70,71] is in ongoing discussions.

Since the large intestine of the gastrointestinal tract plays a major role in the pathogenesis of Stx-caused diseases, the human colon epithelial cell lines Caco-2 and HCT-8 have been and are still globally used cell lines to unravel Stx-mediated damage, based on the fact that both express the Stx receptor Gb3Cer [62,72]. Only limited data are available for Caco-2 and HCT-8 cells regarding the exact structures of their potential Stx-receptor GSLs Gb3Cer and Gb4Cer; the binding specificity or prevalence of Stx towards certain lipoforms of the receptor GSLs; and their suspected association with membrane microdomains, also named as *lipid rafts*. To this end, we performed a detailed analysis covering the missing pieces to round up the picture regarding the fine structures of Stx GSL receptors and their membranous lipid environment in Stx-susceptible human Caco-2 and HCT-8 colon epithelial cell lines.

## 2. Results

In this study, we focused on the identification and structural characterization of GSLs of the two human colon epithelial cell lines Caco-2 and HCT-8, which act as receptor GSLs for Stx2a, the most frequent HUS-associated Stx subtype. Moreover, we probed their association with membrane microdomains using detergent-resistant membranes (DRMs) as *lipid raft*-equivalent supramolecular structures and determined the Stx2a-mediated cellular damage of Caco-2 and HCT-8 cells in comparison to Vero-B4 cells.

### 2.1. Identification of Neutral GSLs of the Globo-Series in Caco-2 and HCT-8 Cells

**Figure 1:**
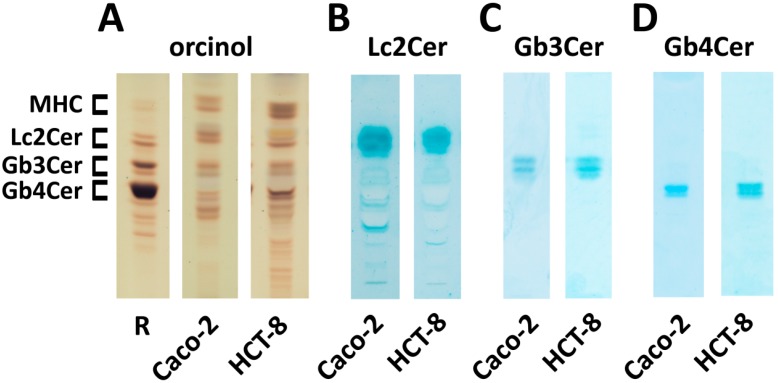
Orcinol stain (**A**); and anti-Lc2Cer (**B**); anti-Gb3Cer (**C**); and anti-Gb4Cer (**D**) overlay assay of TLC-separated neutral GSL preparations from Caco-2 and HCT-8 cell lines. The applied GSL amounts correspond to 5 × 10^6^ cells for the orcinol stain (**A**); 1 × 10^7^ cells for the Lc2Cer (**B**); and 2 × 10^5^ cells for the Gb3Cer (**C**); and the Gb4Cer overlay assay (**D**), respectively. R: 20 µg of reference neutral GSLs from human erythrocytes. MHC, monohexosylceramides.

Neutral GSLs were isolated by anion-exchange chromatography from in vitro-propagated epithelial cells and analyzed by thin-layer chromatography (TLC). The orcinol stain of Figure 1 demonstrates the presence of mono-, di-, tri- and tetrahexosylceramides in both cell lines with proposed monohexosylceramide (MHC), lactosylceramide (Lc2Cer), Gb3Cer and Gb4Cer structures (Figure 1A). A preparation of neutral GSLs from human erythrocytes, which is known to contain the globo-series GSLs Gb3Cer and Gb4Cer, served as reference for assignment of hypothetical GSL structures in the preparations of Caco-2 and HCT-8 cells in the early stage of the investigation. Caco-2 cells exhibit a balanced GSL profile with almost equally stained GSLs, whereas the chromatogram of HCT-8 cells suggests a somewhat higher relative content of MHC and Gb4Cer when compared to Caco-2 cells. Lc2Cer, the precursor GSL of Gb3Cer, was detected in the GSL fractions of both cell lines with a specific anti-Lc2Cer antibody (Figure 1B). Gb3Cer and Gb4Cer were immunochemically detected with an anti-Gb3Cer and an anti-Gb4Cer antibody, as shown in Figure 1C,D, respectively. The slightly diffuse TLC immunostained bands suggest some ceramide heterogeneity of the individual GSLs, which obviously appear as distinct lipoforms in Caco-2 and HCT-8 cells.

### 2.2. Structural Characterization of Sphingolipids from the Neutral GSL Fraction of Caco-2 and HCT-8 Cells

**Figure 2:**
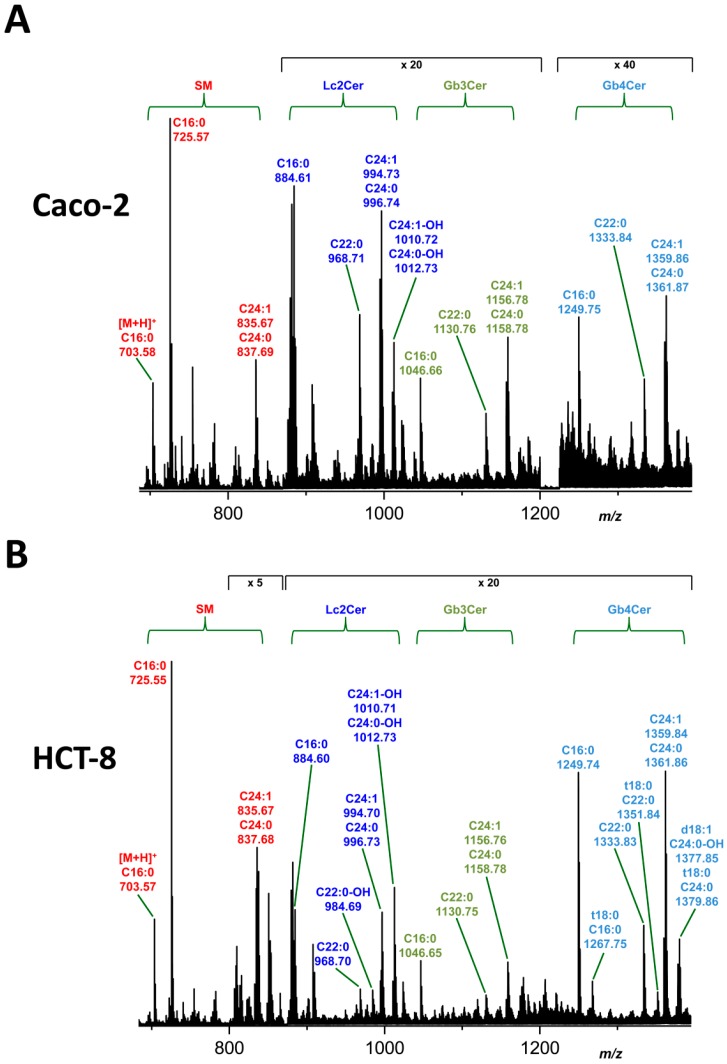
Overview MS^1^ spectra of the neutral GSL fractions of: Caco-2 (**A**); and HCT-8 cells (**B**). The identified di-, tri- and tetrahexosylceramides were assigned to Lc2Cer, Gb3Cer and Gb4Cer deduced from TLC overlay assays (see Figure 1). All GSL species were detected as monosodiated [M + Na]^+^ ions using the positive ion mode and are listed in Table 1 for Caco-2 cells and Table 2 for HCT-8 cells.

The overview (MS^1^) spectrum of the neutral GSL preparation of Caco-2 cells, which was obtained from measurements in the positive ion mode, indicates the presence of Lc2Cer, Gb3Cer and Gb4Cer species all arising as monosodiated [M + Na]^+^ ions as the major GSLs (Figure 2A). The various lipoforms derive from differing ceramide moieties harboring d18:1 sphingenine (sphingosine) as the long chain amino alcohol, which is linked to a C16:0, C22:0 or C24:0/C24:1 fatty acid (Table 1). Two Lc2Cer species were found to carry a hydroxylated fatty acid. In addition to the mentioned GSLs, four sphingomyelin (SM) variants were detected in the spectrum, of which SM with Cer (d18:1, C16:0) at *m*/*z* 703.58 was the only sphingolipid that appeared as protonated [M + H]^+^ ions. Proposed structures were verified by collision-induced (CID) mass spectrometry (not shown). The monohexosylceramides glucosylceramide (GlcCer) and/or galactosylceramide (GalCer) could not be unequivocally determined in the MS^1^ spectrum due to highly abundant SM in the *m*/*z* area of interest (Figure 2A). The differentiation between GlcCer and GalCer requires TLC separation as borate complexes followed by extraction of the analytes from the silica gel and structural characterization by mass spectrometry as shown in the next paragraph.

The positive ion mode MS^1^ spectrum of the neutral GSL preparation of HCT-8 cells revealed Lc2Cer, Gb3Cer and Gb4Cer as major GSLs, which all occur as [M + Na]^+^ ions (Figure 2B). The different lipoforms are due to ceramide heterogeneity with d18:1 sphingenine (sphingosine) long chain base coupled to a C16:0, C22:0 or C24:0/C24:1 fatty acid (Table 2). Of note, four Gb4Cer lipoforms were detected carrying a t18:0 long chain base (“t” stands for “trihydroxylated”) and three Lc2Cer species were identified with a hydroxylated fatty acid. Four SM species were detectable including exceptionally protonated SM (d18:1, C16:0) at *m*/*z* 703.57. Proposed structures were approved by CID mass spectrometry (not shown). The detection of monohexosylceramides GlcCer and/or GalCer was hampered by sphingomyelin ions with similar *m*/*z*-values to the monohexosylceramides (Figure 2B), which had to be separated as borate complexes before MS analysis, as outlined in the next paragraph.

### 2.3. Structural Characterization of GlcCer and GalCer Lipoforms Detected in the Neutral GSL Fraction of Caco-2 and HCT-8 Cells

**Figure 3:**
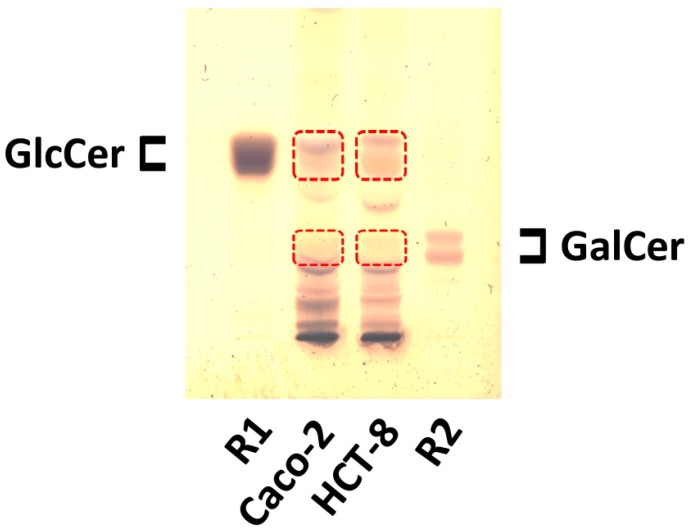
Orcinol stain of the neutral GSLs from Caco-2 and HCT-8 cell lines after TLC separation as borate complexes. Applied GSL amounts correspond to 5 × 10^6^ Caco-2 and HCT-8 cells, respectively. Framed boxes indicate areas from which GSLs of a companion unstained plate were extracted for MS analysis of GlcCer and GalCer of Caco-2 (see Figure 4) and HCT-8 cells (see Figure 5). R1: 20 µg reference GlcCer; R2: 5 µg of reference GalCer.

The monohexosylceramides were separated as borate complexes into GlcCer and GalCer in comparison to reference GlcCer from human Gaucher’s spleen (R1) and GalCer from human brain (R2), respectively (Figure 3). The orcinol-stained bands indicated presence of GlcCer in Caco-2 and HCT-8 cells, whereas GalCer was almost undetectable with orcinol. GalCer seems to be covered by a yellowish compound that interferes by separating at the same position as GalCer on the TLC plate.

**Figure 4:**
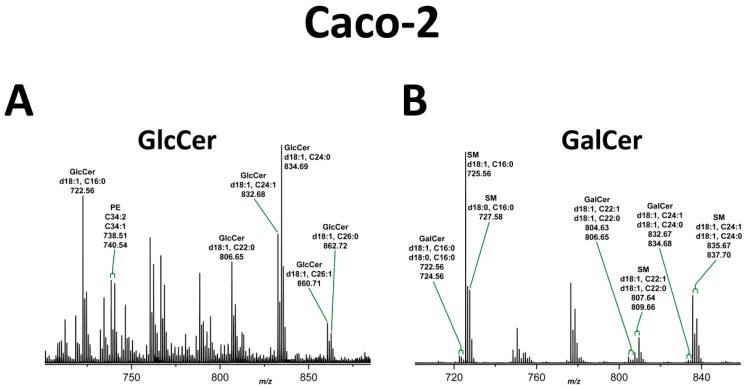
MS^1^ spectra of: GlcCer (**A**); and GalCer species (**B**) of Caco-2 cells. GSL spectra were obtained from silica gel extracts of the GlcCer and GalCer zones after TLC separation of Caco-2 GSLs as borate complexes (see Figure 3). PE (phosphatidylethanolamine) and SM (sphingomyelin): co-extracted lipids. Non-labeled ion signals derive from co-extracted impurities. MS^2^ spectra of: GlcCer (d18:1, C16:0) (**A**); and GalCer (d18:1, C16:0) (**B**) are exemplarily provided in Figure S1A,B, respectively, to demonstrate the structural proof of MS^1^-based proposed structures.

Positive ion mode mass spectrometric analysis of the silica gel extracts obtained from unstained areas at the position of GlcCer and GalCer after TLC separation of the neutral GSL preparations of Caco-2 and HCT-8 cells (see dashed boxes in Figure 3) resulted in the structural characterization of monohexosylceramides from both cell lines. In the GlcCer zone of Caco-2 cells, we detected GlcCer variants harboring Cer (d18:1, C16:0), Cer (d18:1, C22:0) and Cer (d18:1, C24:1/C24:0) as the prominent GlcCer species, accompanied by minor GlcCer variants with Cer (d18:1, C26:1/C26:0), as shown in the overview mass spectrum (Figure 4A). The MS^2^ spectrum of GlcCer (d18:1, C16:0) with the fragmentation scheme is shown in Figure S1A as a representative example for verification of the various GlcCer lipoforms. The MS^1^ spectrum of the extract obtained from the GalCer area revealed minimal amounts of GalCer with Cer (d18:1/d18:0, C16:0), Cer (d18:1, C22:1/C22:0) and Cer (d18:1, C24:1/C24:0) indicated by low abundant ions in the MS^1^ spectrum (Figure 4B). The MS^2^ spectrum obtained by simultaneous CID analysis of GalCer (d18:1/d18:0, C16:0) and the corresponding fragmentation scheme are shown in Figure S1B as a representative example of verified GalCer lipoforms and gives an impressive demonstration of the mass spectrometric power for the analysis of very minor GSLs. Several SM species, which co-separate with GalCer on the TLC plate, were detected as the dominant sphingolipids in the MS^1^ spectrum. They possibly correspond to the yellowish bands observed at the position of GalCer in the orcinol-stained chromatogram of Figure 3.

**Figure 5:**
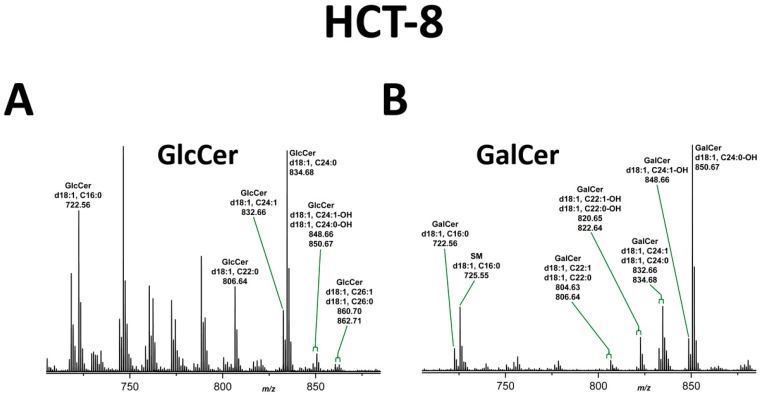
MS^1^ spectra of: GlcCer (**A**); and GalCer species (**B**) of HCT-8 cells. GSL spectra were obtained from silica gel extracts of the GlcCer and GalCer zones after TLC separation of HCT-8 GSLs as borate complexes (see Figure 3). SM (sphingomyelin): co-extracted lipid. Non-labeled ion signals derive from co-extracted impurities. MS^2^ spectra of: GlcCer (d18:1, C16:0) and GlcCer (d18:1, C22:0) (**A**); and GalCer (d18:1, C16:0) and GalCer (d18:1, C24:0-OH) (**B**) are exemplarily provided in Figures S2 and S3, respectively, to demonstrate the structural proof of MS^1^-based proposed structures.

The inspection of the MS^1^ spectrum, which was obtained from the GlcCer zone of TLC-separated neutral GSLs of HCT-8 cells (see Figure 3), revealed the presence of prevalent GlcCer lipoforms built of Cer (d18:1, C16:0), Cer (d18:1, C22:0) and Cer (d18:1, C24:1/C24:0), accompanied by minor GlcCer species with hydroxylated Cer (d18:1, C24:1-OH/C24:0-OH) and Cer (d18:1, C26:1/C26:0) as shown in Figure 5A. MS^2^ spectra of GlcCer (d18:1, C16:0) and GlcCer (d18:1, C22:0) exemplarily show the structural verification by CID analysis in Figure S2A,B, respectively. The MS^1^ spectrum of the extract from the TLC GalCer zone gave evidence for GalCer (d18:1, C24:0-OH) with hydroxylated saturated C24 fatty acid as the predominant GSL species accompanied by less abundant GalCer (d18:1, C24:1-OH) and the non-hydroxylated pendants with Cer (d18:1, C24:1/C24:0) as minor compounds (Figure 5B). Further less abundant ions were indicative for GalCer lipoforms with Cer (d18:1, C22:1-OH/C22:0-OH) and the non-hydroxylated Cer (d18:1, C22:1/C22:0) pendants. Minor GalCer (d18:1, C16:0) and GalCer (d18:1, C24:0-OH) were chosen as representative examples of GalCer lipoforms carrying a hydroxylated fatty acid to demonstrate the structural proof by means of CID measurements together with the fragmentation schemes, as shown in Figure S3A,B, respectively,. The fragment ions obtained by internal cleavage of the ceramide portion clearly evidenced the occurrence of C24:0-OH fatty acid and sphingosine (d18:1) of the dominant GalCer lipoform of HCT-8 cells (Figure S3B). Finally, one SM species arising as low abundant sphingolipid species was found in the overview mass spectrum.

### 2.4. Relative Content of the Various Gb3Cer and Gb4Cer Lipoforms of Caco-2 and HCT-8 Cells

**Figure 6:**
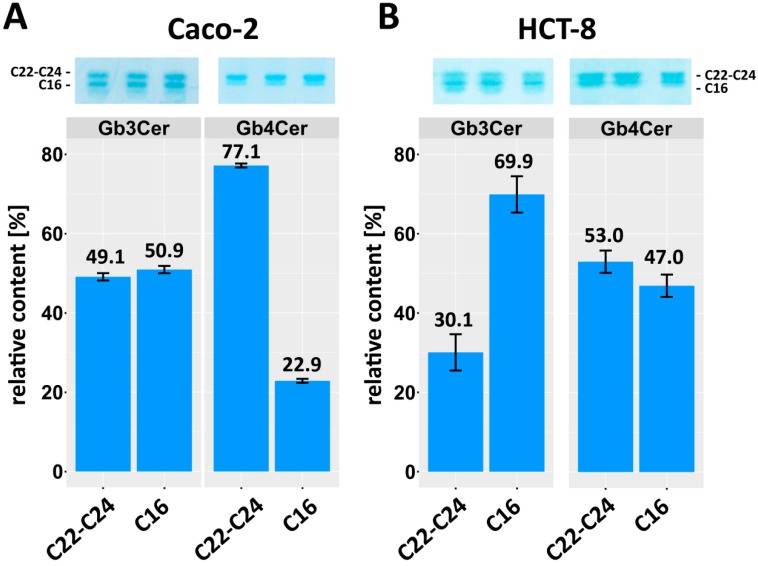
Relative content of Gb3Cer and Gb4Cer with long-chain fatty acids (C22-C24) versus Gb3Cer and Gb4Cer with short-chain fatty acid (C16) of: Caco-2 cells (**A**); and HCT-8 cells (**B**). Immunostained Gb3Cer and Gb4Cer upper bands (C22-C24) and Gb3Cer and Gb4Cer lower bands (C16) of GSL preparations from three biological replicates (above) were scanned and the relative amounts, each normalized to 100%, are shown as bar charts (below) of scanned GSL bands. Applied GSL amounts correspond to 2 × 10^5^ Caco-2 and HCT-8 cells, respectively.

TLC immunopositive Gb3Cer and Gb4Cer bands of the neutral GSL preparations from three independent biological replicates of each cell line were utilized for quantification of the relative content of Gb3Cer with long-chain (C22-C24) and short-chain (C16) fatty acids, which separate in the upper and lower band on the TLC plate, respectively. The Gb3Cer and Gb4Cer bands of Caco-2 and HCT-8 cells shown in Figure 6 (upper panels) were scanned and the relative intensities are shown as bar charts (lower panels) in Figure 6A,B, respectively. An almost equal distribution of 49.1% (±0.9%) Gb3Cer (C22-C24) and 50.9% (±0.9%) Gb3Cer (C16) was determined from the scans for Caco-2 cells (Figure 6A), whereas values of Gb4Cer evidence prevalence of Gb4Cer (C22-C24) with 77.1% (±0.3%) over 22.9% (±0.3%) of Gb4Cer (C16). A strikingly different relative distribution of Gb3Cer and Gb4Cer lipoforms was observed for HCT-8 cells as can be deduced from bar charts depicted in Figure 6B. Here, Gb3Cer (C16) clearly dominates with 69.9% (±4.6%) over 30.1% (±4.6%) Gb3Cer (C22-C24), whereas Gb4Cer exhibits a nearly balanced distribution of 53.0% (±2.8%) of Gb4Cer (C22-C24) and 47.0% (±2.8%) of Gb4Cer (C16). In the following paragraph, we will provide data on those Gb3Cer and Gb4Cer species that are the prevalent receptors for Stx2a based on Stx TLC overlay assay detection.

### 2.5. Identification and Structural Characterization of Stx2a-Binding GSLs

**Figure 7:**
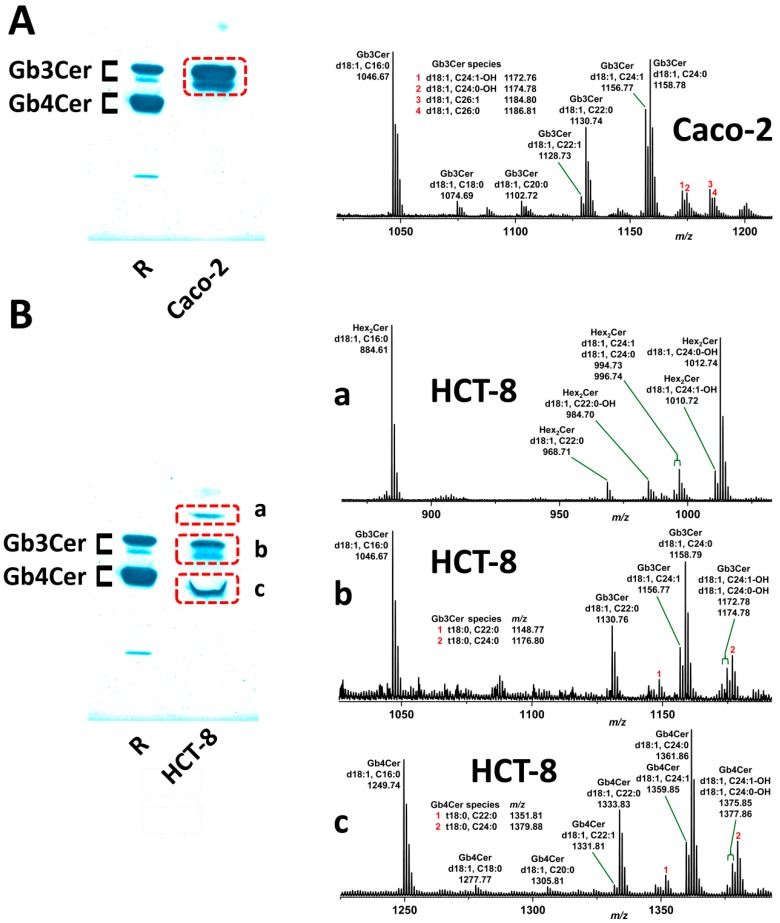
Structures of Stx2a-binding GSLs of: Caco-2 cells (**A**); and HCT-8 cells (**B**), determined by Stx2a TLC overlay assays (left panels) and mass spectrometric analysis (right panels). Caco-2 cells (**A**) exhibit a couple of Gb3Cer variants as the dominant Stx2a-binding structures. HCT-8 cells (**B**) harbor one or more Stx2a-binding GSLs within the cluster of: the detected Hex_2_Cer species (**a**); and several Stx2a-binding Gb3Cer (**b**); and Gb4Cer (**c**) variants. Applied GSL amounts for the overlay assays correspond to 5 × 10^6^ Caco-2 and HCT-8 cells, respectively. R: 4 µg of reference neutral GSLs from human erythrocytes with Gb4Cer as the prevalent and Gb3Cer as the less abundant GSL (see orcinol stain in Figure 1A). (**A**) The MS^2^ spectrum and the corresponding fragmentation scheme of Gb3Cer (d18:1, C26:1 (compound **3**) are shown in Figure S4 to exemplarily demonstrate the structural proof of MS^1^-based proposed structures from Caco-2 cells. (**B**) The same holds true for MS^2^ spectra of: Hex_2_Cer (d18:1, C24:0-OH) (**a**); Gb3Cer (d18:1, C24:1/C24:0) (**b**); and Gb4Cer (d18:1, C24:0-OH)/Gb4Cer (t18:0, C24:0) (**c**), which were obtained from HCT-8 cells and are provided in Figures S5–S7, respectively.

Stx2a-binding Gb3Cer and Gb4Cer species were detected using TLC-separated GSLs of Caco-2 and HCT-8 cells. For this purpose, chromatograms were overlaid with Stx2a, followed by incubation with an anti-Stx2 antibody and the corresponding alkaline phosphatase-labeled secondary antibody. The enzyme reaction was visualized with an indolyl-phosphate dye as a blue precipitate (for details refer to the “Materials and Methods”). This direct Stx2a-mediated receptor detection revealed a strongly positive Gb3Cer double band for Caco-2 cells (Figure 7A, left), which consisted of various Gb3Cer lipoforms as shown by electrospray ionization mass spectrometry (ESI-MS) analysis (Figure 7A, right). Gb3Cer (d18:1, C16:0), Gb3Cer (d18:1, C22:0) and Gb3Cer (d18:1, C24:1/C24:0) were the prevalent species detected in the MS^1^ spectrum, which were also detectable in the previous overview mass spectrum obtained from the total GSL fraction (see Table 1). In addition to these prominent Gb3Cer species, some minor variants were detected in the spread Gb3Cer spectrum obtained from the silica gel extract of the Stx2a-positive GSL bands shown in Figure 7A, including minute Gb3Cer lipoforms harboring C18:0, C20:0, C22:1, C26:1, C26:0 and hydroxylated C24:1-OH and C24:0-OH fatty acids. The MS^2^ spectrum of Gb3Cer (d18:1, C26:1) (Figure 7A, compound **3**) is shown in Figure S4 as an impressive example for the MS-based structural characterization of very minor GSL species.

The Stx2a overlay assay of the GSLs from HCT-8 cells exhibited a more complex overlay picture indicating Stx2a-binding GSLs at the position of dihexosylceramide above Gb3Cerand Gb4Cer (Figure 7B, left). The MS^1^ spectrum of the Stx2a-positive dihexosylceramide area (Figure 7B, right, subpanel a) might contain one or more Stx2a-binding dihexosylceramides such as galabiosylceramide (Galα1-4Galβ1-1Cer) or other dihexosylceramides with terminal Galα1-4-configuration, but most likely as minor constituents. Due to the abundant presence of Lc2Cer in HCT-8 cells (see Figure 1 and Figure 2 and Table 2), no definite decision can be made for Stx2a-binding dihexosylceramides, because the most prominent species of the spectrum might correspond to underlying Lc2Cer species. However, they do not bind to Stx2a, but separate on the same position in the silica gel layer of the chromatogram. Therefore, dihexosylceramides are assigned as Hex_2_Cer structures in the MS^1^ spectrum in the subpanel a of Figure 7B, because Galβ1-4Glcβ1-1Cer (Lc2Cer) and, e.g., Galα1-4Glcβ1-1Cer, cannot be distinguished by ESI mass spectrometry. The MS^2^ spectrum of Hex_2_Cer (d18:1, C24:0-OH) is given in Figure S5. Further examples of structural verifications of Stx2a-binding GSLs of HCT-8 cells are presented for Gb3Cer (d18:1, C24:1/C24:0) from subpanel b of Figure 7B in Figure S6 and for Gb4Cer (d18:1, C24:0-OH)/Gb4Cer (t18:0, C24:0) from subpanel c of Figure 7B in Figure S7.

### 2.6. Distribution of Gb3Cer and Gb4Cer to DRM and NonDRM Fractions

**Figure 8:**
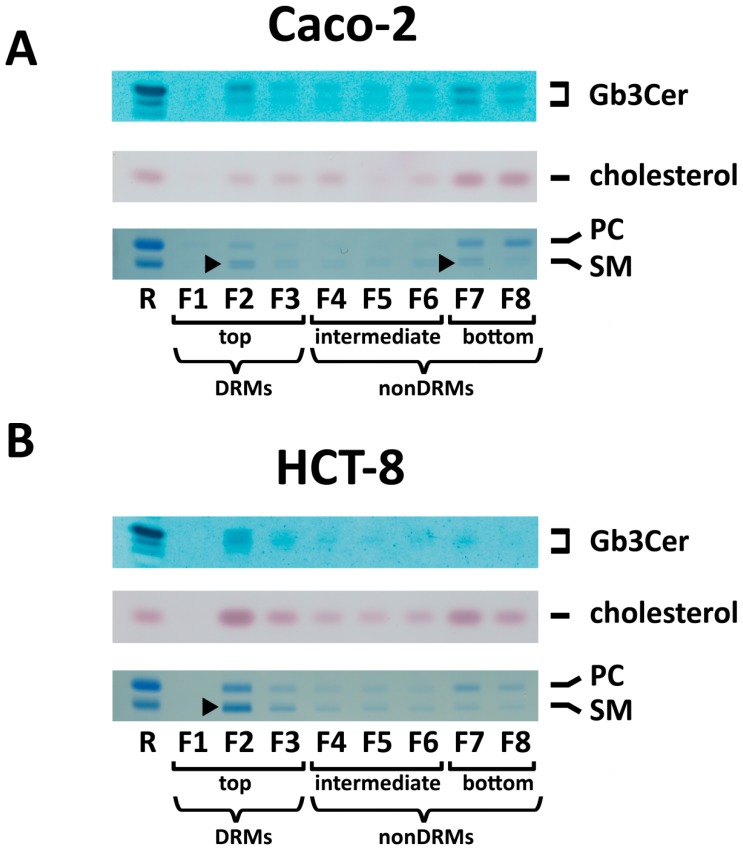
Distribution of Gb3Cer, cholesterol and the phospholipids sphingomyelin (SM) and phosphatidylcholine (PC) in sucrose gradient fractions of: Caco-2 cells (**A**); and HCT-8 cells (**B**). (**A**) Anti-Gb3Cer TLC overlay assay and detection of TLC-separated cholesterol of lipid extracts from gradient fractions F1 to F8 correspond to 2.5 × 10^6^ cells, respectively, and those for phospholipid staining to 1 × 10^7^ cells. R: references for Gb3Cer detection are 2 µg of neutral GSLs from human erythrocytes, 0.5 µg of cholesterol for staining with manganese(II) chloride and 5 µg of SM and 4 µg of PC for molybdenum blue staining of phospholipids. (**B**) Anti-Gb3Cer TLC overlay assay and cholesterol detection correspond to 1 × 10^7^ cells, respectively, and those for phospholipid staining to 3 × 10^7^ cells. R: references for Gb3Cer detection are 2 µg of neutral GSLs from human erythrocytes, 0.5 µg of cholesterol and 5 µg of SM and 4 µg of PC for staining of phospholipids. The arrowheads mark: balanced distribution of SM to F2 and F7 of Caco-2 cells (**A**); and preferred occurrence of SM in F2 of HCT-8 cells (**B**). DRMs, detergent-resistant membranes.

To get some hints on possible association of Stx receptors with *lipid rafts*, we compared the distribution of the major Stx receptor Gb3Cer to detergent-resistant membranes (DRMs) and non–DRM fractions with that of cholesterol and phospholipids. Figure 8 shows examples of antibody-mediated detection of Gb3Cer (overlay assays) and stains of cholesterol and the phospholipids PC and SM of TLC-separated lipid extracts from sucrose gradient fractions obtained from Caco-2 (Figure 8A) and HCT-8 cells (Figure 8B). The analyzed gradient fractions F1 to F8 were grouped into DRM (F1 to F3) and non–DRM fractions (F4 to F8), which were further subgrouped into intermediate (F4 to F6) and bottom fractions (F7 to F8). Gb3Cer was found for Caco-2 cells to clearly distribute to the F2 fraction of DRMs (top fractions), but shows considerable appearance in the bottom fractions with some preference to fraction F7 of non–DRMs and trace amounts in the intermediate fractions F4 to F6 as well (Figure 8A, upper panel). SM, although only weakly detectable, exhibited almost equal distribution to F2 and F7 (Figure 8A, lower panel), whereas cholesterol showed obvious preference for the bottom fractions F7 and F8 (Figure 8A, middle panel). A typical example of TLC-analyzed lipids in sucrose gradient fractions of HCT-8 cells is provided in Figure 8B, indicating preference of Gb3Cer for top fraction F2 of DRMs (Figure 8B, upper panel), almost balanced presence of cholesterol in F2 and F7 (Figure 8B, middle panel) and prevalence of SM in F2 (Figure 8B, lower panel).

**Figure 9:**
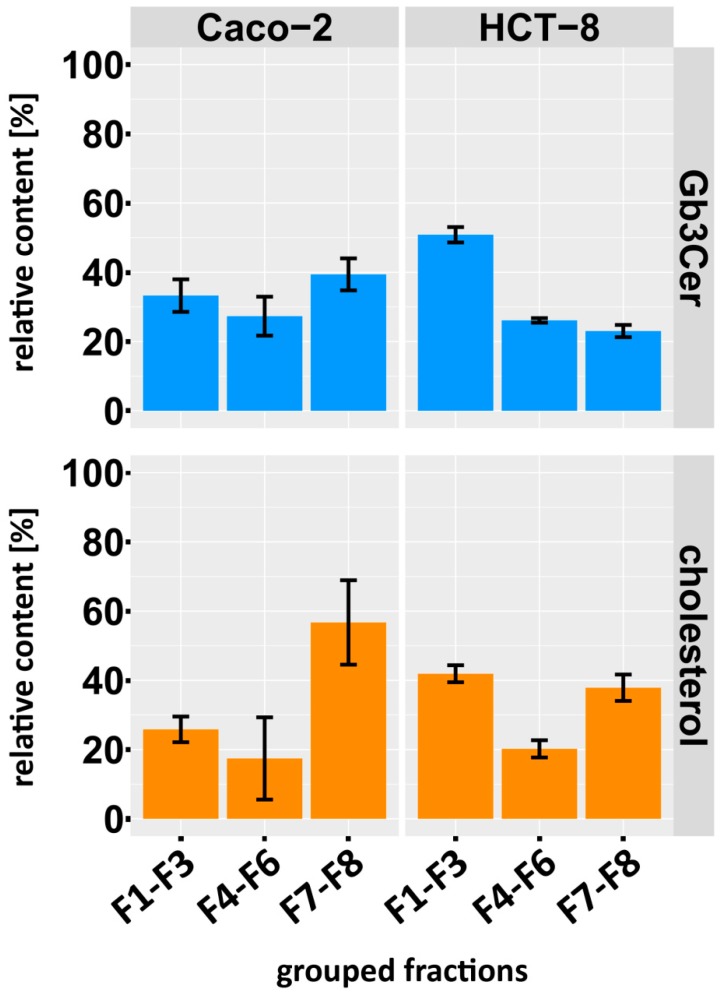
Relative content of Gb3Cer and cholesterol in grouped sucrose gradient fractions of Caco-2 and HCT-8 cells. Values of the F1–F8 gradient fractions were grouped into top fractions (F1–F3), intermediate fractions (F4–F6) and bottom fractions (F7–F8) (see Figure 8). Each distribution of grouped fractions was normalized to 100% and averaged values obtained from TLC scans of three independent biological replicates are depicted.

A summary of the average relative values obtained from quantitative TLC scans of the individual lipid bands of Gb3Cer and cholesterol from three independent biological replicates is depicted as a bar chart in Figure 9. Values of grouped fractions F1–F3 (DRMs, top fractions), F4–F6 (intermediate fractions) and F7–F8 (bottom fractions) revealed an almost balanced Gb3Cer distribution to the three classical sucrose gradient zones in case of Caco-2 cells (Figure 9, upper left panel) with 33.3 ± 4.7% in F1–F3, 27.3 ± 5.6% in F4–F6 and 39.4 ± 4.6% in F7–F8, whereas HCT-8 exhibited a distinct preference for Gb3Cer with 50.9 ± 2.2% in F1–F3 compared to 26.1 ± 0.7% in F4–F6 and 23.0% ± 1.8% in F7–F8, indicating *lipid raft* association of the major Stx receptor GSL (Figure 9, upper right panel). The cholesterol distribution of Caco-2 cells did not correlate with the Gb3Cer distribution, owing to its tendency to preferably occur with 56.7 ± 12.2% in the F7–F8 bottom fractions (Figure 9, lower left panel) compared to 25.8 ± 3.7% in the F1–F3 top fractions and 17.4 ± 11.8% in the F4–F6 intermediate fractions. An opposite discrepancy was found for HCT-8 cells, where cholesterol distributed almost equally to the F1–F3 fractions with 41.9 ± 2.5% and to the F7–F8 fractions with 37.9 ± 3.8% when compared to intermediate F4–F6 fractions with 20.2 ± 2.5% (Figure 9, lower right panel), while Gb3Cnon–DRM er showed preference for the grouped F1–F3 fractions.

### 2.7 Stx2a-Mediated Cellular Damage of Caco-2 and HCT-8 Cells

**Figure 10:**
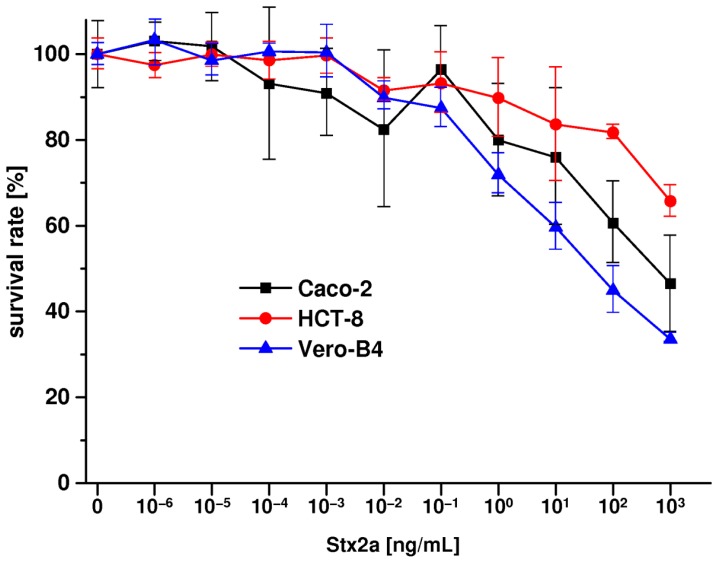
Cytotoxic action of Stx2a towards Caco-2, HCT-8 and Vero-B4 cell lines. Stx2a-mediated cytotoxicity was determined using the crystal violet assay and absorption values obtained from Stx2a-treated cells are depicted as percentage of untreated cells, which were set to 100% survival rate. Results represent the means (standard deviations) of four-fold determinations. Vero-B4 cells were employed as positive control and reference cell culture.

Stx2a was applied to Caco-2 and HCT-8 and Vero-B4 reference cell cultures with increasing concentrations from 10^−6^ ng/mL (≡1 fg/mL) up to 10^3^ ng/mL (≡1 µg/mL) as shown in Figure 10. Notably, the three cell lines were cultured under serum-free conditions to exclude any serum-derived artifacts. At low concentrations in the range from 10^−6^ ng/mL to 10^−1^ ng/mL (≡1 fg/mL to 100 pg/mL), Stx2a did not cause a significant growth inhibitory effect on Caco-2 and HCT-8 cells. Sensitivity became apparent at a concentration of 1 ng/mL whereby Caco-2 cells showed somewhat higher susceptibility towards Stx2a when compared to HCT-8 cells (Figure 10). The survival rates upon Stx2a exposure decreased in a concentration-dependent manner to 75.9 ± 15.8% and 83.6 ± 13.2% at 10 ng/mL, 60.6 ± 9.5% and 81.8 ± 1.7% at 100 ng/mL and to 46.5 ± 11.2% and 65.7 ± 3.7% at 1000 ng/mL for Caco-2 and HCT-8 cells, respectively. The relatively high standard deviations, especially in case of Caco-2 cells, can be attributed to somewhat inhomogeneous growth behavior of the cells, which tend to grow in clusters before reaching the confluent stage. Vero-B4 cells revealed an approximately 20% higher sensitivity when compared to Caco-2 cells with a final survival rate of 33.5 ± 1.1% at the highest Stx2a concentration applied. Collectively, both human colon epithelial cell lines exhibited significant susceptibility towards Stx2a being little less sensitive when compared to Vero-B4 cells. Vero cells were used, because they are known as a highly Stx-sensitive cell line, which is commonly used as the gold standard in Stx cytotoxicity assays.

## 3. Discussion

The structural diversity of ubiquitously distributed amphipathic GSLs, comprising hundreds of different oligosaccharides and tens of diverse ceramide structures [73,74,75], represents a potential for numerous biological functions [76] including the initiation of a bacterium-host interplay mediated by protein–carbohydrate interaction [77]. For instance, uropathogenic *E. coli* and the gastric colonizer *Helicobacter pylori* have developed strategies to adhere to certain host glycoconjugates via adhesins and to enter niches in human hollow organs such as the bladder and the stomach, respectively [78,79,80,81]. Furthermore, various bacterial toxins have evolved that are released by pathogens and exploit GSLs of host target cells as attachment sites [82]. Stxs do belong to the group of GSL-binding toxins, which are delivered into the gut by STEC during infection [83,84,85]. The various lipoforms of GSLs, based on structural heterogeneity of the fatty acid and the long-chain base of the ceramide moiety, and their association with *lipid rafts* are supposed as a functional requirement of binding, internalization and retrograde transportation of bacterial toxins to their intracellular sites of action [86,87,88,89,90,91,92]. Therefore, we performed a precise GSL analysis of Stx GSL receptors of human Caco-2 and HCT-8 colon epithelial cell lines including analysis of their membrane lipid environment and determining Stx2a-mediated cellular damage compared to the reference Vero-B4 cell line.

Caco-2 cells have been previously shown to respond upon Stx exposure with the production of cytokines [93] and with apoptosis and inhibition of protein synthesis related to binding of Stx to Gb3Cer [61,94], however, leaving the structures of Stx receptor GSLs largely unexplored in these studies. The presence of the globo-series neutral GSLs Gb3Cer and Gb4Cer has been determined in extracts of Caco-2 cells by TLC overlay assays using metabolically labeled P-fimbriated *E. coli* [95]. Exposure of Caco-2 cells to butyrate, a known transcriptional regulator of differentiation genes in many cell types, has been reported to promote the expression of the Stx receptors Gb3Cer and Gb4Cer and toxin sensitivity [72]. Interestingly, a plant-based recombinant secretory IgA antibody with binding specificity towards the Stx B-subunit, which has been developed as an edible therapeutic antibody for oral immunotherapy, was shown to neutralize the cytotoxicity of Stx1(a) towards butyrate-treated Caco-2 cells [96]. The internalization of the B-subunit of Stx1 could be significantly lowered by *lipid raft* disruption by cholesterol depletion. This finding suggests that *lipid rafts* are necessary for toxin uptake across the apical membrane of Caco-2 cells, although *lipid raft* disintegration did not affect the amount of bound Stx1(a) B-subunit [97]. Anyway, Stx-susceptible Caco-2 cells harbor *lipid raft*-associated Stx receptors reasoned from studies where Stx1(a) and Stx2(a) were found to bind to detergent-insoluble microdomains, a term equivalently used for DRMs as denoted in our study [98]. The analysis of *raft*-type membrane microdomains, which correspond to DRMs as prepared in our study, revealed preliminary data on the lipid composition of such membrane clusters of Caco-2 cells suggesting presence of cholesterol, sphingomyelin and GSL in DRM subsets [99]. The ceramide lipid anchor of GSLs has been precisely determined for mono-, di-, tri- and tetrahexosylceramides of Caco-2 cells in a previous study by Tanaka and co-workers using matrix-assisted laser desorption/ionization time-of-flight mass spectrometry (MALDI-TOF MS) [100]. Emphasis was placed in this study on the analysis of ceramide alterations upon exposure of the cells to different partial pressure of oxygen (normoxia, hypoxia and reoxygenation). Aberrant ceramide hydroxylation of the tri- and tetrahexosylceramides has been detected when switching Caco-2 cells from normoxic to hypoxic conditions and subsequent reoxygenation [100]. Ceramide hydroxylation adds a further facet of modulating the biophysical properties of Gb3Cer and Gb4Cer that might lead to altered binding of Stx [87]. This should be kept in mind as an interesting aspect for future investigations. However, we did not detect hydroxylated Gb3Cer and Gb4Cer lipoforms in the GSL preparation of Caco-2 cells in our study. This could be explained by different cell culture conditions, because we performed serum-free cultivation without adding fetal calf serum to in vitro propagated cells in order to avoid possible influence of xenogenous serum on GSL expression [101]. Thus, since Caco-2 cells are globally used under different aspects of Stx-caused cellular response, our study may help to better understand Stx-mediated biological effects on this human colon-derived cell line. A cell type-specific receptor profile and the extent of *lipid raft* association of membranous GSL receptors and their lipid environment in microdomains of the plasma membrane should be of concern for the cellular response on Stx exposure.

HCT-8 cells have been so far less intensively investigated with respect to Stx-caused cellular effects when compared to globally used Caco-2 cells. HCT-8 cells have been found being sensitive towards Stx1(a) treatment, which resulted in cell death that was associated with caspase 3 cleavage and internucleosomal DNA fragmentation [102]. Both Stx1a and Stx2a (formerly named Stx1 and Stx2 in the literature and by us; for nomenclature refer to Scheutz et al. [28]) were found to bind to the human colonic epithelial HCT-8 cell line, which does express the globo-series neutral GSLs Gb3Cer, less abundantly, and Gb4Cer, more abundantly [62]. From this report, it could be concluded that Gb3Cer may be present in small quantities in human colonic epithelia, where it may compete for Stx binding with the more abundantly expressed GSL Gb4Cer. Findings of Zumbrun and colleagues are in line with our GSL analysis giving evidence for high abundance of Gb4Cer and somewhat less abundance of Gb3Cer in HCT-8 cells. Furthermore, we could show that Gb4Cer was recognized by Stx2a in addition to Gb3Cer. The TLC overlay assays clearly evidenced attachment of Stx2a to Gb4Cer, which is known to be recognized less efficiently by Stx1a and Stx2a [30]. This effect has been shown being considerably pronounced when mixtures of Gb3Cer and Gb4Ger with GlcCer, GalCer or Lc2Cer were used in ELISA approaches [103]. Of note, Stx-mediated injury of HCT-8 cells could be enhanced 20- to 60-fold by genetic manipulation of the cells that resulted in an increase of the cell surface Gb3Cer content [62]. Moreover, Gb3Cer levels on butyrate-exposed HCT-8 cells increased 10-fold and exhibited, when maintained in butyrate, an 1000-fold higher sensitivity to Stx1(a) intoxication [104]. On the other hand, pretreatment of HCT-8 cells with cholesterol-depleting methyl-β-cyclodextrin significantly decreased flagellin-mediated invasion by Shiga-toxigenic *E. coli* O113:H21 indicating a functional role for *lipid rafts* in the invasion mechanism [105]. Recently, we have used the neutral GSL fraction of HCT-8 cells in a methodological approach for probing MALDI-MS imaging of TLC-separated GSLs, i.e., detection of GSLs directly from the TLC plate without preceding extraction from the silica gel layer [106]. Thus, the HCT-8 cell line represents a suitable model cell line being in use to explore the molecular and cellular mechanisms underlying Stx-mediated damage of the human colon epithelium. Our data on Stx receptors of HCT-8 cells including knowledge of the full structures of the various Gb3Cer and Gb4Cer lipoforms might be of benefit for all researchers, which are involved in EHEC research aimed at unraveling the complicated host–pathogen interplay by using this cell line.

Common features with respect to GSL-binding Stx of serum-free cultivated Caco-2 and HCT-8 cells are the expression of similar Gb3Cer and Gb4Cer lipoforms comprising chiefly ceramide moieties composed of sphingosine (d18:1) and C16:0, C22:0 or C24:0/C24:1 fatty acid. The most significant difference between the two cell lines was the prevalence of Gb3Cer with C16 fatty acid in HCT-8 and Gb4Cer with C22-C24 fatty acids in Caco-2 cells. In contrast to the Caco-2 and HCT-8 cell lines, the human colon epithelial T84 cell line has been reported to lack any globo-series GSLs and to resist Stx-induced disruption of protein biosynthesis and apoptosis [58,61,72,107]. Although lacking Gb3Cer (and Gb4Cer) structures, T84 cells are capable to internalize Stx1(a) and Stx2(a) and to transport the toxins to the endoplasmic reticulum [61,107] and/or to translocate the toxins across T84 monolayers [107]. These results suggest a general mechanism by which bacterial toxins that lack specific intestinal receptors can penetrate the intestinal epithelial barrier.

Notably, Gb3Cer and Gb4Cer species carrying saturated C16:0, C22:0 or C24:0 fatty acid as well as the unsaturated C24:1 fatty acid were the dominant GSLs of the globo-series expressed by human colon Caco-2 and HCT-8 epithelial cells. These are the same previously identified in various human endothelial cell lines [52,54] as well as primary human umbilical vein endothelial cells [48] and primary human endothelial cells of the brain [56] and the kidney [57]. Questions about the biological significance of this conserved repertoire of globo-series neutral GSLs in human endothelial and colon epithelial cells with presumed impact on Stx attachment, uptake and retro-translocation remain yet unanswered. However, studies on lipid bilayer model membranes spiked with Gb3Cer species composed of ceramides carrying fatty acids with different acyl chain structure gave evidence that the fatty acid chain length and saturation level (saturated versus unsaturated) as well as hydroxylation may affect many Gb3Cer involving processes such as the formation of a membrane liquid ordered phase and endocytic membrane invaginations upon toxin binding [33,108,109,110,111,112]. Of note, Gb3Cer variants harboring an unsaturated acyl chain did cause the formation of tubular invaginations in contrast to Gb3Cer with saturated acyl chain [33]. This effect has also been described for ganglioside GM1 species carrying an unsaturated fatty acid being the only GM1 lipoform that could sort GM1-binding cholera toxin efficiently from the plasma membrane to the *trans*-Golgi network and the endoplasmic reticulum [113]. It is therefore tempting to speculate that the Gb3Cer and Gb4Cer variants with unsaturated fatty acid, namely Gb3Cer (d18:1, C24:1) and Gb4Cer (d18:1, C24:1), detected as the prevalent globo-series lipoforms in the mass spectra of all as yet precisely analyzed human endothelial cell lines [43,45,52,54] and primary endothelial cells [48,56,57] as well as the human colonic Caco-2 and HCT-8 cell lines investigated in this study, might be the receptor species being mainly responsible for subcellular sorting of Stxs. Since infections of EHEC expressing Stx2a are associated with a higher risk for the development of HUS [114,115] and the most dangerous and fatal outbreak strains such as EHEC O157:H7 [2,116,117,118,119] or O104:H4 [120,121] do express Stx2a, we used this Stx subtype in our study to determine its cytotoxic activity on human colon Caco-2 and HCT-8 epithelial cell lines. The Vero-B4 cell line served as an Stx-susceptible reference cell line, which has been previously analyzed in detail with respect to its Stx receptor profile and Stx susceptibility [32]. The cellular sensitivity of Caco-2 cells was somewhat more pronounced when compared to HCT-8 cells, which were less sensitive. Vero-B4 cells exhibited the highest Stx susceptibility in comparative cytotoxicity assays as expected. Nevertheless, it should be re-emphasized that both colon epithelial cell lines showed significant cellular damage upon Stx exposure, giving again evidence that indeed not only the human vascular endothelium but also the human colon epithelium represents a potential target for the clinically most relevant Stx2a subtype.

## 4. Conclusions

To further our understanding of the complex mechanisms underlying binding and internalization of the various Stx-subtypes, research directed at blocking cellular attachment and ensuing uptake of Stxs and thereby neutralizing their cytotoxic activity may help to develop new therapeutic strategies with the ultimate aim to successfully combat EHEC-caused infections. With our study, we want to add a further piece to completing the puzzle of the protein–carbohydrate Stx-host interaction at the initial stage of human EHEC infections.

## 5. Materials and Methods

### 5.1. Cultivation of Caco-2, HCT-8 and Vero-B4 Cell Lines and Stx2a Cytotoxicity Assay

The Caco-2 cell line and the Vero-B4 reference cell line were obtained from the German Collection of Microorganisms and Cell Cultures (DSMZ, Braunschweig, Germany; DSMZ No. ACC 169 and No. ACC 33, respectively); the HCT-8 cell line was purchased from the American Type Culture Collection (ATCC, Manassas, VA, USA; ATCC No. CCL-244). The cells were cultivated in a humidified atmosphere at 37 °C with 5% CO_2_ in chemically defined serum-free OptiPRO^TM^ SFM medium (Gibco Life Technologies Corporation, Paisley, UK; catalogue No. 12309-019) supplemented with L-glutamine to 4 mM final concentration prior to use. The attachment dependent cell lines grew as monolayers and were routinely passaged every 2–3 days using 0.25% Trypsin-EDTA (Invitrogen, Karlsruhe, Germany; Cat. No. 25200) before reaching the confluent stage. Appropriate amounts of Caco-2 and HCT-8 cells were produced in 175 cm^2^ tissue culture flasks (Greiner Bio-One, Frickenhausen, Germany) for subsequent isolation of GSLs from total cells and for the preparation of sucrose density gradient fractions as previously described [32,54,122].

The crystal violet assay was employed for determination of Stx2a-mediated cellular damage as previously described [55,69,122]. Briefly, 24 h after seeding the cells into microtiter plate wells, subconfluent cells were exposed for 1 h to Stx2a dilutions in serum-free cell culture medium starting with the highest concentration of 1 µg/mL (final volume of 200 µL). Stx-free cell culture medium served as a control. Toxin treatment was stopped by removal of the Stx-containing medium, which was replaced by fresh medium. After incubation for another 48 h the cell cultures were stopped and the crystal violet assay was performed as previously described in detail [55,69,122]. The percentage of live cells was calculated from the absorbance of destained cells monitored at 570 nm using a microtiter plate reader (OpsysMR absorbance reader; Dynex, Berlin, Germany). Results represent the means ± standard deviations (SD) of quadruplicate determinations, and are portrayed as percentage values of untreated control cells.

### 5.2. Isolation of Lipids and Purification of GSLs from Cultured Cell Lines 

Lipids were isolated from total cells of three independently produced cell culture batches, respectively, of confluent grown cells as previously described [32,54,69]. Briefly, lipid isolation was started with methanol as the first extraction solvent. The methanolic suspension was centrifuged and the sediment was successively extracted with chloroform/methanol (1/2, *v*/*v*), chloroform/methanol (1/1, *v*/*v*) and chloroform/methanol (2/1, *v*/*v*). The pooled supernatants were evaporated and coextracted alkali-labile phospholipids and triglycerides were removed by mild saponification. Neutral GSLs were isolated by anion-exchange column chromatography as previously described [32,54,69] using DEAE-Sepharose CL-6B (GE Healthcare, Munich, Germany) according to standard procedures [123,124] and finally dissolved in chloroform/methanol (2/1, *v*/*v*).

### 5.3. Preparation of Sucrose Density Gradient Fractions by Ultracentrifugation

Sucrose density gradient fractions were prepared according to the classical procedure published by Brown and Rose [125] with minor modifications as previously described [25,32,54]. In short, confluent grown Caco-2 and HCT-8 cells were disrupted in lysis buffer and the cell debris was separated by gentle centrifugation (400× *g*), followed by short ultracentrifugation (150,000× *g*) of the supernatant to separate membranes from the cytoplasm. The membrane sediment was solubilized in 1% Triton X-100 buffer and mixed in the same proportion with 85% sucrose. The resulting 42.5% sucrose solution was then overlayed with a discontinuous sucrose gradient of 30% and 5% sucrose. After ultracentrifugation (200,000× *g*) eight fractions of 1.5 mL each were collected one by one from top of the gradient: three DRM-associated top fractions (F1–F3) and five non–DRM fractions beneath (F4–F8). The non–DRM fractions were further subgrouped into intermediate (F4–F6) and bottom fractions (F7–F8).

### 5.4. Isolation of Lipids from Sucrose Density Gradient Fractions

Sucrose was removed from the gradient fractions by dialysis against deionized water at 4 °C for 2 days. For phospholipid analysis, 0.5 mL of each fraction was freeze dried, solubilized under sonication in chloroform/methanol (2/1, *v*/*v*) and adjusted to defined volumes adequate to 1 × 10^5^ cells/µL. For GSL and cholesterol analysis, 0.5 mL freeze-dried aliquots of the gradient fractions were dissolved in 0.5 mL 1 N NaOH and incubated for 1 h at 37 °C to saponify phospholipids and triglycerides followed by neutralization with HCl [25,32,54]. After desalting by dialysis and lyophilization, the extracts were dissolved in chloroform/methanol (2/1, *v*/*v*) and adjusted to a concentration corresponding to 1 × 10^5^ cells/µL.

### 5.5. Lipid References, Antibodies and Stx2a

A preparation of neutral GSLs from human erythrocytes harboring the Stx receptor GSLs Gb3Cer (Galα1-4Galβ1-4Glcβ1-1Cer) and Gb4Cer (GalNAcβ1-3Galα1-4Galβ1-4Glcβ1-1Cer) [126,127,128] was used as positive control for anti-Gb3Cer and anti-Gb4Cer as well as the Stx2a TLC overlay assays as described in previous publications [25,55,56,57,65,75,129]. A GlcCer fraction from human Gaucher’s spleen was purchased from Sigma-Aldrich (St. Louis, MO, USA; Cat. No. G-9884) and a GalCer fraction was prepared from human brain according to standard procedures [123,130]. The nomenclature of GSLs follows the IUPAC-IUB recommendations 1997 [131]. Cholesterol (Sigma Aldrich, Steinheim, Germany; Cat. No. C8667) and a phospholipid standard mixture containing phosphatidylcholine (PC) and sphingomyelin (SM) were used as references for lipid analysis of sucrose gradient fractions as previously described [25,32,54,132].

Polyclonal chicken IgY anti-Lc2Cer, anti-Gb3Cer and anti-Gb4Cer antibodies with previously described specificities [24,65,129,133] were employed for TLC overlay assays. Bacterial cell culture supernatant from *E. coli* serotype O111:H- (strain 03-06016, Stx2a) [30] was used for detection of Stx2a receptors in TLC overlay assays and for purification of Stx2a, which has been previously described for purification of Stx2 from *E. coli* strain C600(933W) [122]. Mouse anti-Stx2 antibody (clone VT 135/6-B9, 2.75 mg/mL) was purchased from SIFIN GmbH (Berlin, Germany). Secondary alkaline phosphatase (AP)-conjugated affinity-purified polyclonal rabbit anti-chicken IgY (code 303-055-033) and goat anti-mouse IgG (code 115-055-003) antibodies were from Dianova (Hamburg, Germany).

### 5.6. Thin-Layer Chromatography and Lipid Staining

For thin-layer chromatography (TLC), lipid preparations were applied to glass-backed high-performance TLC plates precoated with silica gel 60 (HPTLC plates, size 10 cm × 10 cm, thickness 0.2 mm, No. 1.05633.0001; Merck, Darmstadt, Germany) using an automatic sample applicator (Linomat 5, CAMAG, Muttenz, Switzerland). Neutral GSLs were chromatographed in chloroform/methanol/water (120/70/17, each by vol.) and stained with orcinol. Specific separation of GlcCer and GalCer was achieved as borate complexes in alkaline solvent composed of chloroform/methanol/water/32% NH_4_OH (65/25/4/0.5, each by vol.) as previously described [24,134]. Phospholipids were chromatographed in chloroform/methanol/isopropanol/triethylamine/0.25% aqueous KCl (30/9/25/18/6, each by vol.) and stained with molybdenum blue Dittmer-Lester reagent [135,136]. Cholesterol was stained upon TLC separation in chloroform/acetone (96/4, *v*/*v*) with manganese(II)chloride [54,137].

### 5.7. TLC Overlay Assay and Lipid Semiquantification

TLC overlay assays were carried out with polyclonal chicken anti-Lc2Cer, anti-Gb3Cer and anti-Gb4Cer and with Stx2a as previously described [24,25,54,133,138]. Briefly, the silica gel layer was fixed after GSL separation with poly (isobutylmethacrylate) (Plexigum P28, Darmstadt, Germany), followed by incubation with 1:2000 diluted primary antibodies in 1% (*w*/*v*) bovine serum albumin (BSA) in phosphate-buffered saline (PBS). The Stx2a-containing bacterial supernatant was used undiluted; the anti-Stx2 antibody was applied in 1:1000 dilution and the secondary AP-conjugated antibodies were used as 1:2000 dilutions (both in 1% BSA in PBS) as previously described [25,30,69,127,133,138]. Bound secondary antibodies were detected with 0.05% (*w*/*v*) 5-bromo-4-chloro-3-indolyl phosphate *p*-toluidine salt (Roth, Karlsruhe, Germany) in glycine solution (pH 10.4), which generates a blue precipitate at sites of antibody binding on the TLC plate. The relative content of TLC-separated immunostained Gb3Cer and Gb4Cer bands and manganese(II)chloride stained cholesterol bands was determined semiquantitatively by densitometry using a CD 60 scanner (Desaga, Heidelberg, Germany, software ProQuant^®^, version 1.06.000) in reflectance mode at a wavelength of 630 nm (Gb3Cer and Gb4Cer) and 365 nm (cholesterol) with light beam slit dimensions of 0.02 mm × 4 mm. The relative contents of immunostained and cholesterol bands of sucrose gradient fractions were determined using Photoshop CS5 (Adobe, Dublin, Ireland).

### 5.8. Mass Spectrometry of GSLs

Mass spectrometry analysis of GSLs was performed by nano electrospray ionization mass spectrometry (nanoESI MS) using a SYNAPT G2-S mass spectrometer (Waters, Manchester, UK) equipped with a Z-spray source. Dried aliquots of GSL preparations from total cells were dissolved in chloroform/methanol (1/4, *v*/*v*) and analyzed in the positive ion sensitivity mode. The source settings were: temperature 80 °C, capillary voltage 0.8 kV, sampling cone voltage 20 V, and offset voltage 50 V. Proposed GSL structures were confirmed using low energy collision-induced dissociation (CID) experiments (MS^2^). For this purpose the GSL precursor ions were selected in the quadrupole analyzer, ion mobility separation was employed (wave velocity 700–800 m/s, wave height 40 V, nitrogen gas flow rate 90 mL/min, helium gas flow rate 180 mL/min) and subsequent fragmentation was performed in the transfer cell using collision energies of 70 to 100 eV (E_lab_). For MS analysis of monohexosylceramides, the silica gel at the position of GalCer and GlcCer was scratched off the glass plate and GSLs were extracted from the silica gel as previously described [24,139]. Structures of individual GSLs were deduced from CID spectra following the nomenclature introduced by Domon and Costello [140,141] for the assignment of the fragment ions obtained by MS^2^ analysis.

## Figures and Tables

**Table 1:** Major GSLs and sphingolipids of Caco-2 cells determined by mass spectrometry combined with TLC immunodetection. ^a^

Compound ^b^	Ceramide	Formula	*m/z*_exp_	*m/z*_calc_
SM ([M + H]^+^)	d18:1, C16:0	C_39_H_80_N_2_O_6_P	703.58	703.5754
SM	d18:1, C16:0	C_39_H_79_N_2_O_6_PNa	725.57	725.5573
SM	d18:1, C24:1	C_47_H_93_N_2_O_6_PNa	835.67	835.6669
SM	d18:1, C24:0	C_47_H_95_N_2_O_6_PNa	837.69	837.6825
Lc2Cer	d18:1, C16:0	C_46_H_87_NO_13_Na	884.61	884.6075
Lc2Cer	d18:1, C22:0	C_52_H_99_NO_13_Na	968.71	968.7014
Lc2Cer	d18:1, C24:1	C_54_H_101_NO_13_Na	994.73	994.7171
Lc2Cer	d18:1, C24:0	C_54_H_103_NO_13_Na	996.74	996.7327
Lc2Cer	d18:1, C24:1-OH	C_54_H_101_NO_14_Na	1010.72	1010.7120
Lc2Cer	d18:1, C24:0-OH	C_54_H_103_NO_14_Na	1012.73	1012.7276
Gb3Cer	d18:1, C16:0	C_52_H_97_NO_18_Na	1046.66	1046.6603
Gb3Cer	d18:1, C22:0	C_58_H_109_NO_18_Na	1130.76	1130.7542
Gb3Cer	d18:1, C24:1	C_60_H_111_NO_18_Na	1156.78	1156.7699
Gb3Cer	d18:1, C24:0	C_60_H_113_NO_18_Na	1158.78	1158.7855
Gb4Cer	d18:1, C16:0	C_60_H_110_N_2_O_23_Na	1249.75	1249.7397
Gb4Cer	d18:1, C22:0	C_66_H_122_N_2_O_23_Na	1333.84	1333.8336
Gb4Cer	d18:1, C24:1	C_68_H_124_N_2_O_23_Na	1359.86	1359.8493
Gb4Cer	d18:1, C24:0	C_68_H_126_N_2_O_23_Na	1361.87	1361.8649

^a^ For TLC immunodetection of Lc2Cer, Gb3Cer and Gb4Cer refer to Figure 1; ^b^ all sphingolipids were detected in the positive ion mode as monosodiated [M+Na]^+^ ions with the exception of [M + H]^+^ ions of SM (d18:1, C16:0) at *m*/*z* 703.58 as indicated.

**Table 2:** Major GSLs and sphingolipids of HCT-8 cells determined by mass spectrometry combined with TLC immunodetection. ^a^

Compound ^b^	Ceramide	Formula	*m*/*z*_exp_	*m*/*z*_calc_
SM ([M + H]^+^)	d18:1, C16:0	C_39_H_80_N_2_O_6_P	703.57	703.5754
SM	d18:1, C16:0	C_39_H_79_N_2_O_6_PNa	725.55	725.5573
SM	d18:1, C24:1	C_47_H_93_N_2_O_6_PNa	835.67	835.6669
SM	d18:1, C24:0	C_47_H_95_N_2_O_6_PNa	837.68	837.6825
Lc2Cer	d18:1, C16:0	C_46_H_87_NO_13_Na	884.60	884.6075
Lc2Cer	d18:1, C22:0	C_52_H_99_NO_13_Na	968.70	968.7014
Lc2Cer	d18:1, C22:0-OH	C_52_H_99_NO_14_Na	984.69	984.6963
Lc2Cer	d18:1, C24:1	C_54_H_101_NO_13_Na	994.70	994.7171
Lc2Cer	d18:1, C24:0	C_54_H_103_NO_13_Na	996.73	996.7327
Lc2Cer	d18:1, C24:1-OH	C_54_H_101_NO_14_Na	1010.71	1010.7120
Lc2Cer	d18:1, C24:0-OH	C_54_H_103_NO_14_Na	1012.73	1012.7276
Gb3Cer	d18:1, C16:0	C_52_H_97_NO_18_Na	1046.65	1046.6603
Gb3Cer	d18:1, C22:0	C_58_H_109_NO_18_Na	1130.75	1130.7542
Gb3Cer	d18:1, C24:1	C_60_H_111_NO_18_Na	1156.76	1156.7699
Gb3Cer	d18:1, C24:0	C_60_H_113_NO_18_Na	1158.78	1158.7855
Gb4Cer	d18:1, C16:0	C_60_H_110_N_2_O_23_Na	1249.74	1249.7397
Gb4Cer	t18:0, C16:0	C_60_H_112_N_2_O_24_Na	1267.75	1267.7502
Gb4Cer	d18:1, C22:0	C_66_H_122_N_2_O_23_Na	1333.83	1333.8336
Gb4Cer	t18:0, C22:0	C_66_H_124_N_2_O_24_Na	1351.84	1351.8441
Gb4Cer	d18:1, C24:1	C_68_H_124_N_2_O_23_Na	1359.84	1359.8493
Gb4Cer	d18:1, C24:0	C_68_H_126_N_2_O_23_Na	1361.86	1361.8649
Gb4Cer	t18:0, C24:1	C_68_H_126_N_2_O_24_Na	1377.85	1377.8598
Gb4Cer	t18:0, C24:0	C_68_H_128_N_2_O_24_Na	1379.86	1379.8598

^a^ For TLC immunodetection of Lc2Cer, Gb3Cer and Gb4Cer refer to Figure 1; ^b^ all sphingolipids were detected in the positive ion mode as monosodiated [M + Na]^+^ ions with the exception of [M + H]^+^ ions of SM (d18:1, C16:0) at *m*/*z* 703.57 as indicated.

## Supplementary Materials

The following are available online at www.mdpi.com/2072-6651/9/11/338/s1, Figure S1: MS^2^ spectra and corresponding fragmentation schemes of GlcCer (d18:1, C16:0) (**A**) and GalCer (d18:1/d18:0, C16:0) (**B**) obtained from Caco-2 cells, Figure S2: MS^2^ spectra and corresponding fragmentation schemes of GlcCer (d18:1, C16:0) (**A**) and GlcCer (d18:1, C22:0) (**B**) obtained from HCT-8 cells, Figure S3: MS^2^ spectra and corresponding fragmentation schemes of GalCer (d18:1, C16:0) (**A**) and hydroxylated GalCer (d18:1, C24:0-OH) (**B**) obtained from HCT-8 cells, Figure S4: MS^2^ spectrum and corresponding fragmentation scheme of Gb3Cer (d18:1, C26:1) detected in the Stx2a-binding GSL fraction of Caco-2 cells (see Figure 7A, compound **3**), Figure S5: MS^2^ spectrum and corresponding fragmentation scheme of Hex_2_Cer (d18:1, C24:0-OH) detected in the Stx2a-binding GSL fraction of HCT-8 cells (see Figure 7B, panel a), Figure S6: MS^2^ spectrum and corresponding fragmentation scheme of Gb3Cer (d18:1, C24:1/C24:0) detected in the Stx2a-binding GSL fraction of HCT-8 cells (see Figure 7B, panel b), Figure S7: MS^2^ spectrum and corresponding fragmentation schemes of Gb4Cer (d18:1, C24:0)/Gb4Cer (t18:0, C24:0) detected in the Stx2a-binding GSL fraction of HCT-8 cells (see Figure 7B, panel c).
