# Supplementary material for: Shiga Toxin Glycosphingolipid Receptors in Human Caco-2 and HCT-8 Colon Epithelial Cell Lines
Source: Toxins (Basel). 2017 Oct 25;9(11):338. doi: 10.3390/toxins9110338 (PMC5705953; doi:10.3390/toxins9110338)
Supplement: Supplementary file 1 [file toxins-09-00338-s001.pdf]

Supplementary Materials: Shiga Toxin Glycosphingolipid Receptors in Human Caco-2 and HCT-8 Colon Epithelial Cell Lines

Ivan U. Kouzel, Gottfried Pohlentz, Julia S. Schmitz, Daniel Steil, Hans-Ulrich Humpf, Helge Karch and Johannes Müthing

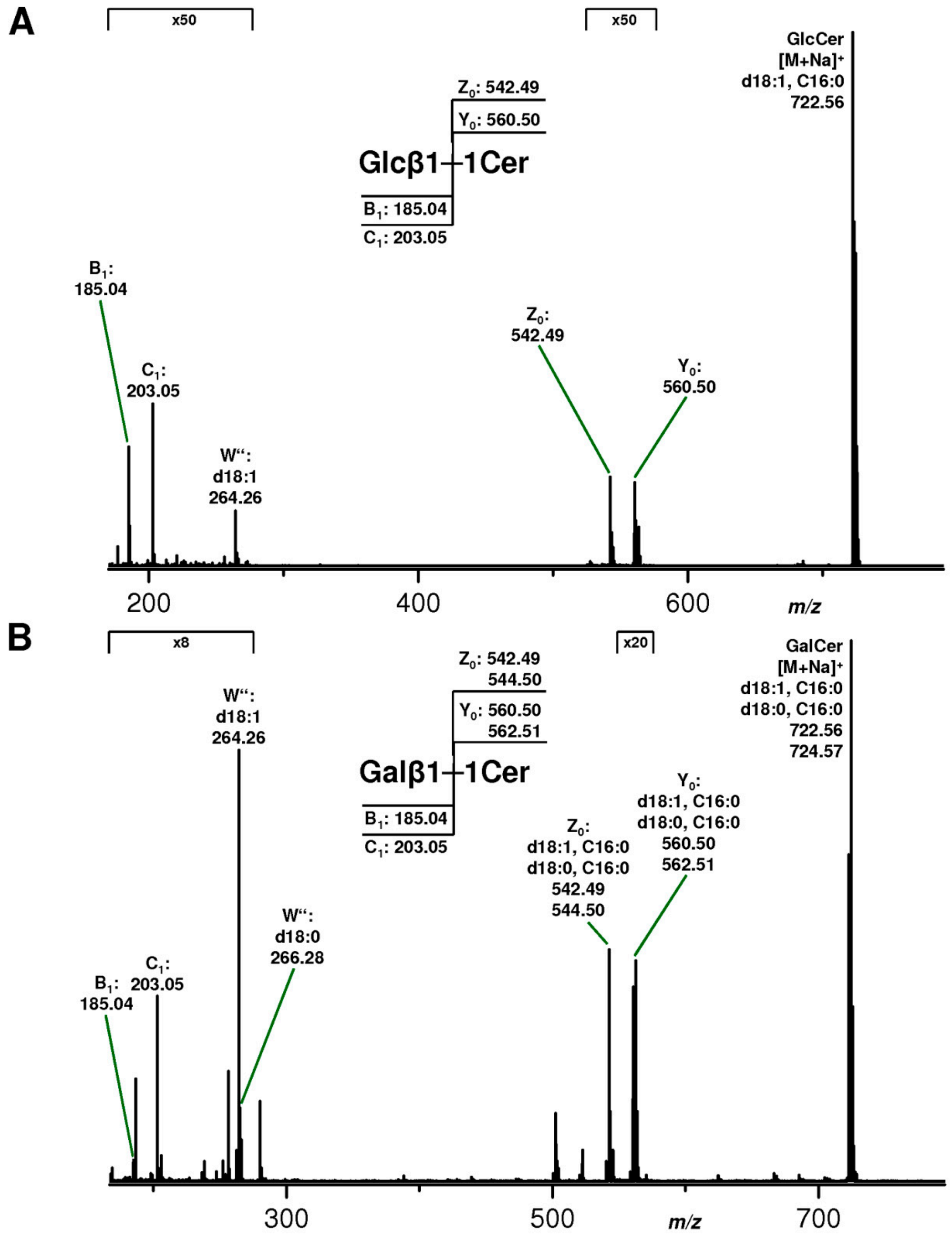

Figure S1. MS^2 spectra and corresponding fragmentation schemes of GlcCer (d18:1, C16:0) (**A**) and GalCer (d18:1/d18:0, C16:0) (**B**) obtained from Caco-2 cells. Non-labeled fragment ion signals derive from coionized impurities.

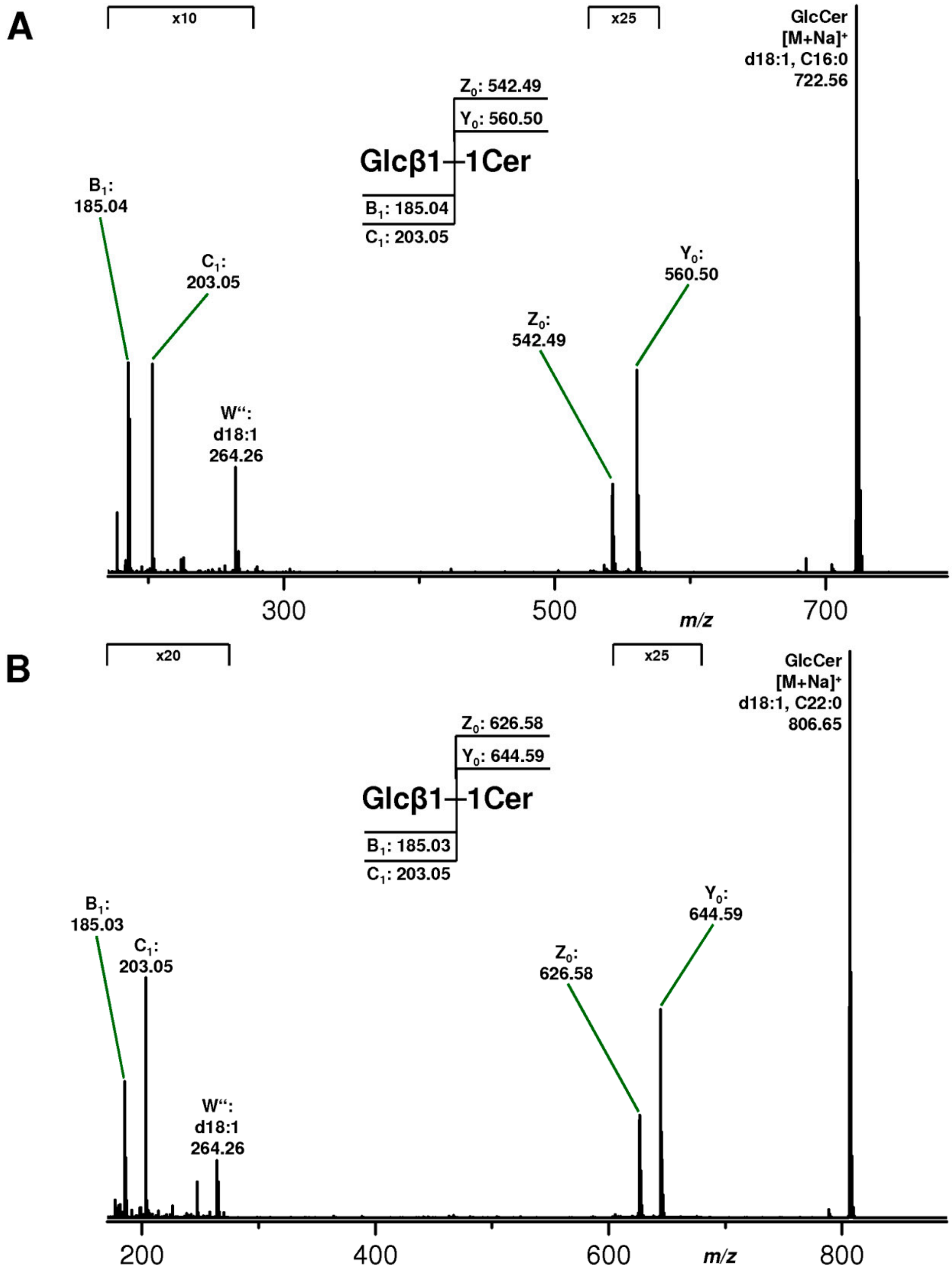

Figure S2. MS^2 spectra and corresponding fragmentation schemes of GlcCer (d18:1, C16:0) (**A**) and GlcCer (d18:1, C22:0) (**B**) obtained from HCT-8 cells. Non-labeled fragment ion signals derive from coionized impurities.

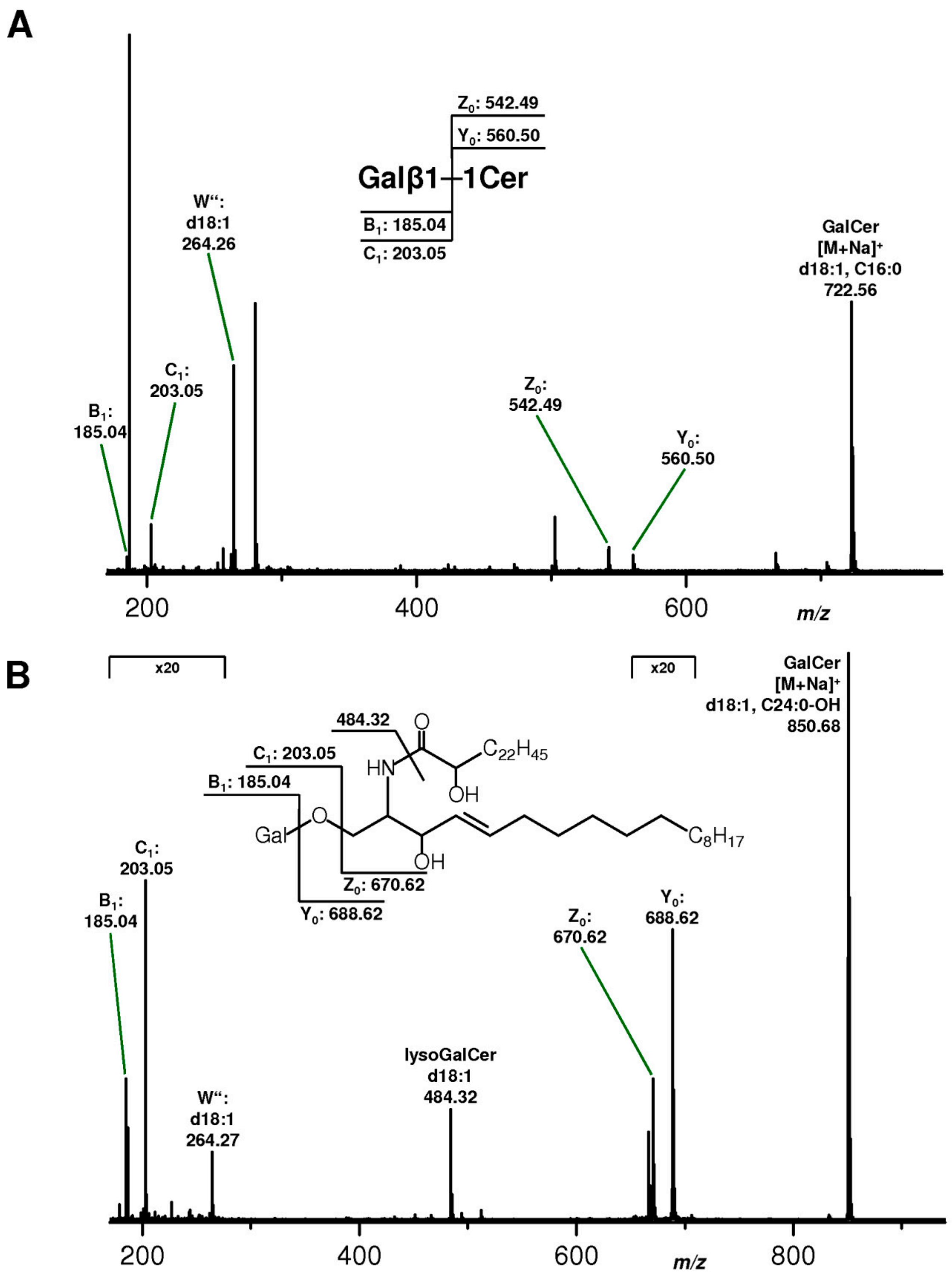

Figure S3. MS^2 spectra and corresponding fragmentation schemes of GalCer (d18:1, C16:0) (**A**) and hydroxylated GalCer (d18:1, C24:0-OH) (**B**) obtained from HCT-8 cells. Non-labeled fragment ion signals derive from coionized impurities.

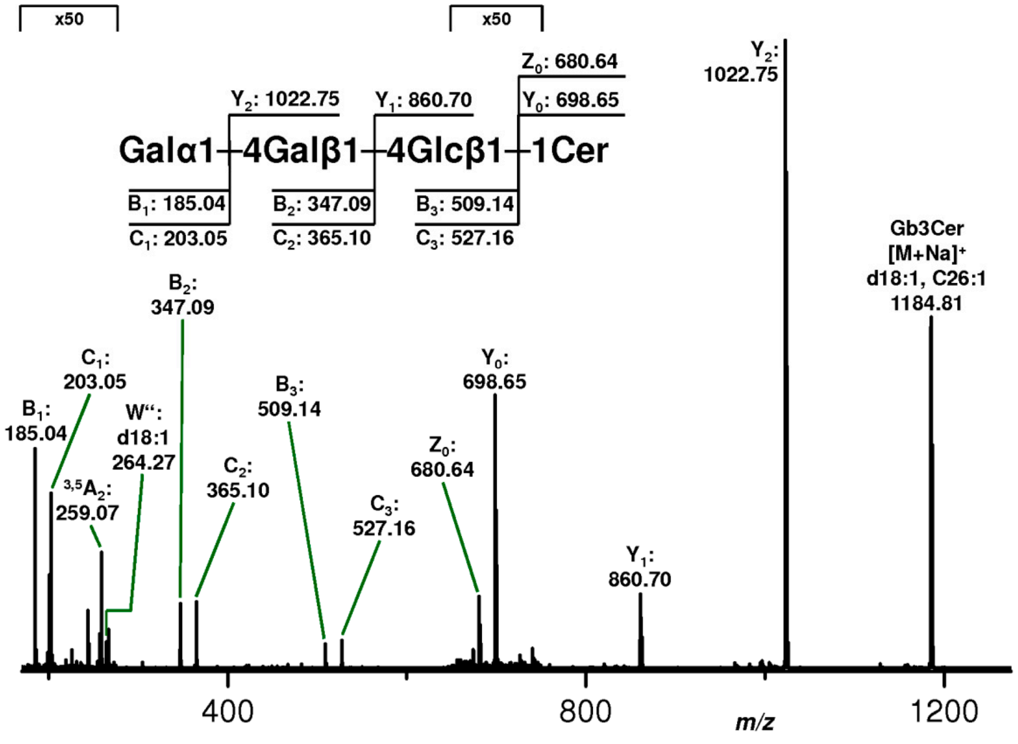

Figure S4. MS^2 spectrum and corresponding fragmentation scheme of Gb3Cer (d18:1, C26:1) detected in the Stx2a-binding GSL fraction of Caco-2 cells (see Figure 7A, compound 3). Non-labeled fragment ion signals derive from coionized impurities.

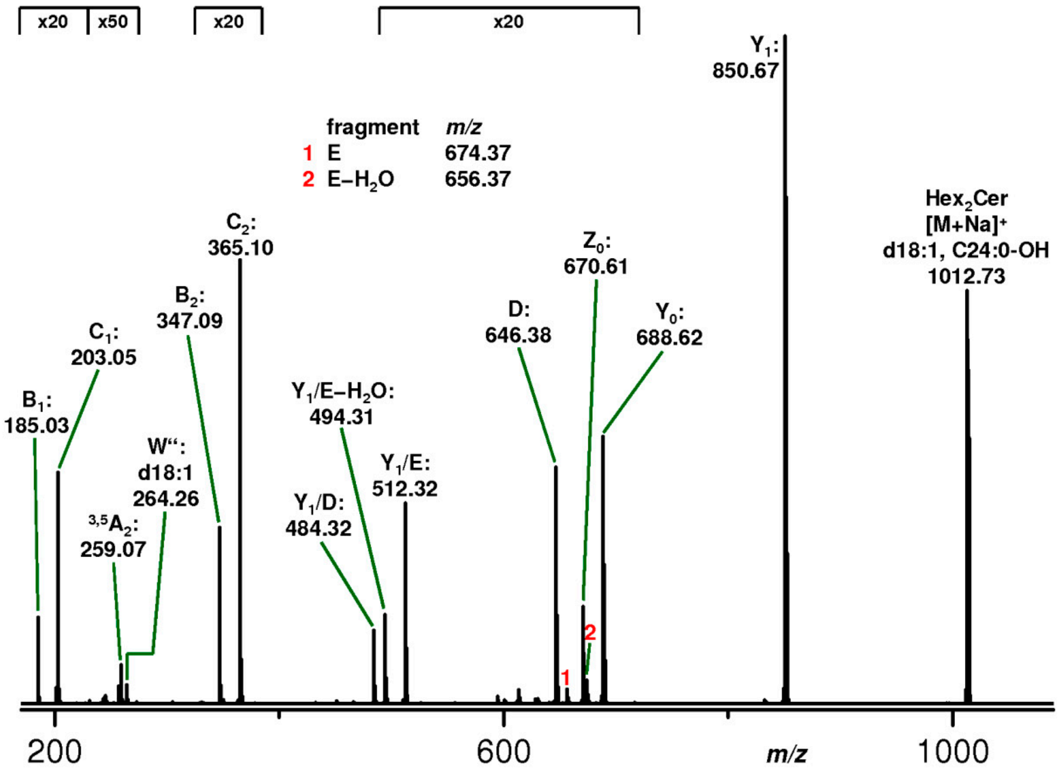

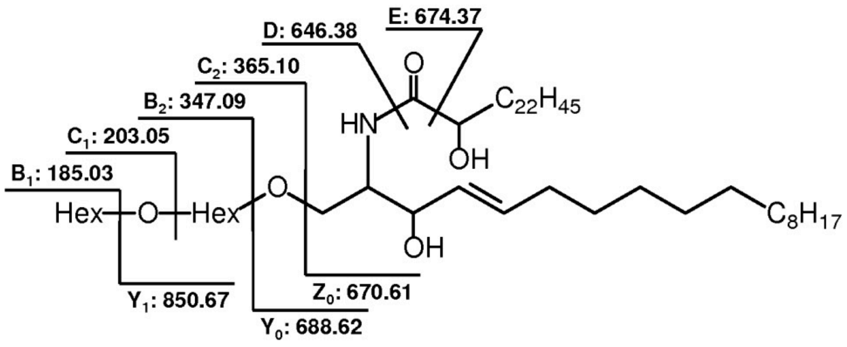

Figure S5. MS^2 spectrum and corresponding fragmentation scheme of Hex_2Cer (d18:1, C24:0-OH) detected in the Stx2a-binding GSL fraction of HCT-8 cells (see Figure 7B, panel a). Non-labeled fragment ion signals derive from coionized impurities.

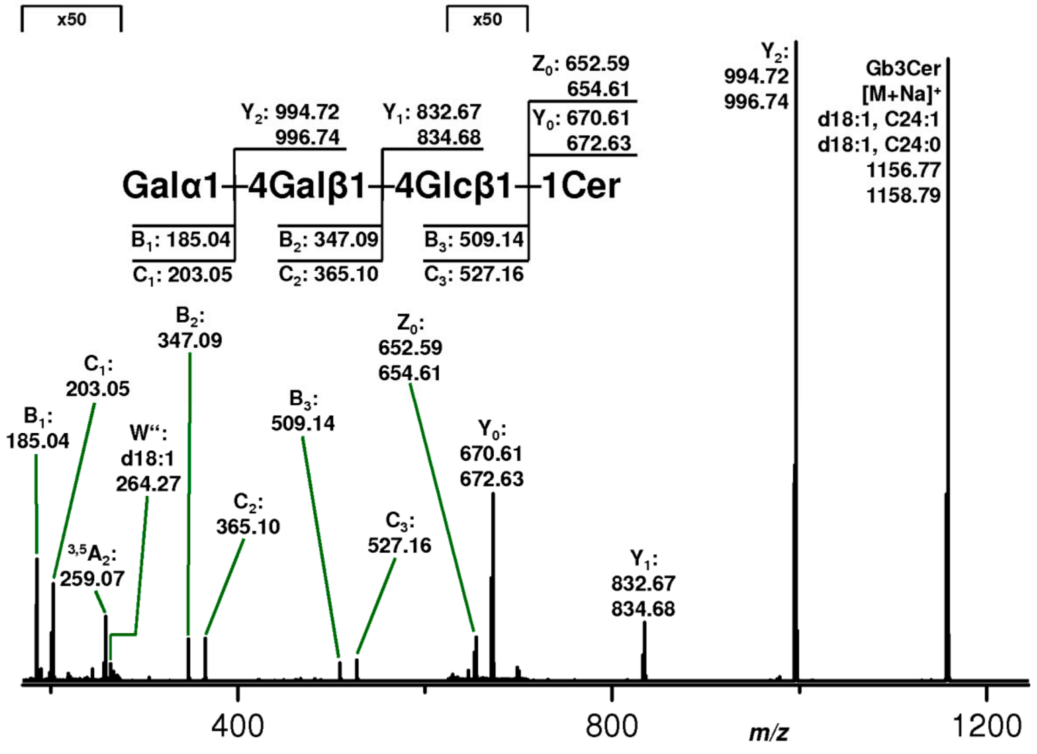

Figure S6. MS^2 spectrum and corresponding fragmentation scheme of Gb3Cer (d18:1, C24:1/C24:0) detected in the Stx2a-binding GSL fraction of HCT-8 cells (see Figure 7B, panel b). Non-labeled fragment ion signals derive from coionized impurities.

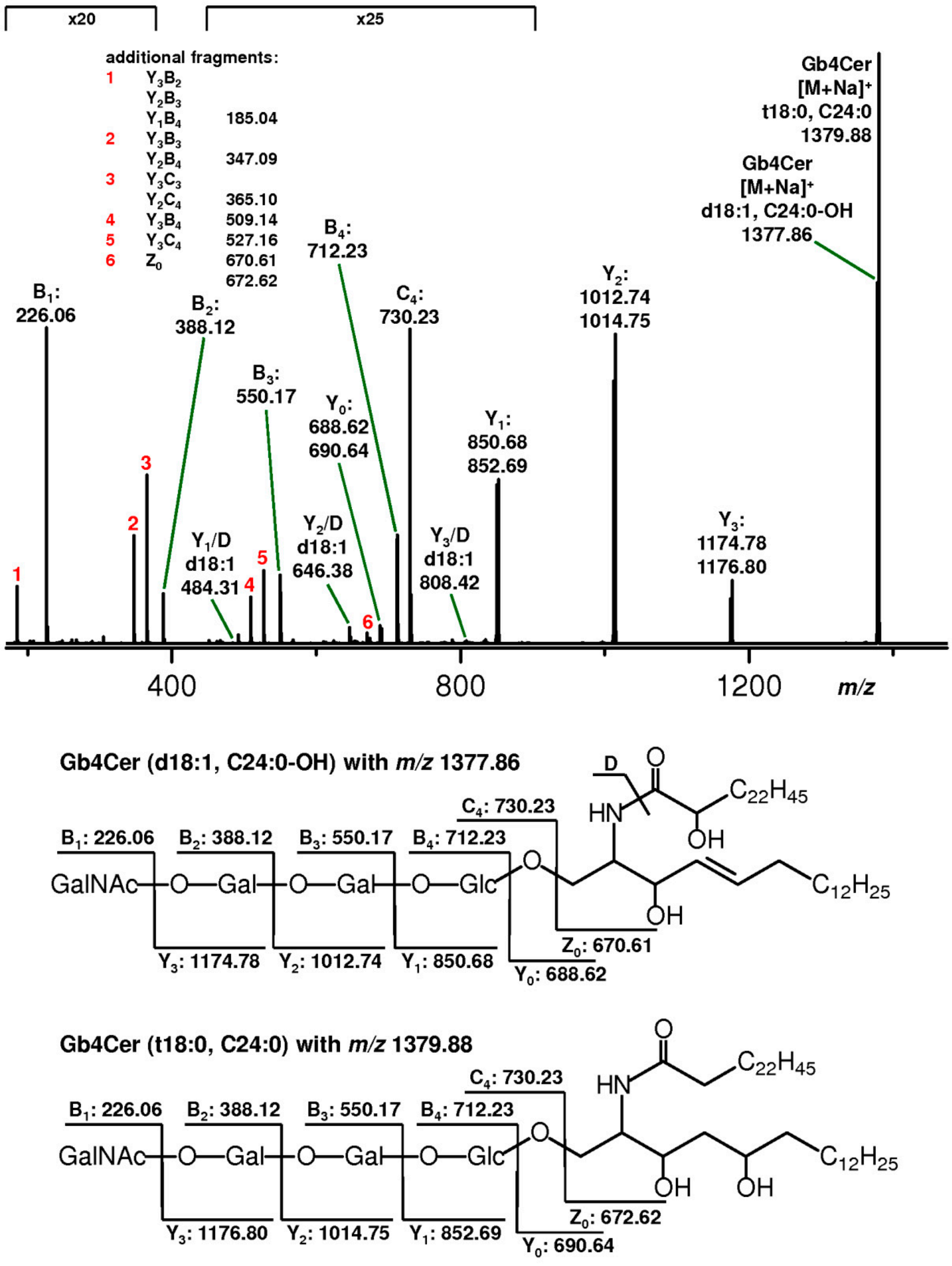

Figure S7. MS[2] spectrum and corresponding fragmentation schemes of Gb4Cer (d18:1, C24:0-OH)/Gb4Cer (t18:0, C24:0) detected in the Stx2a-binding GSL fraction of HCT-8 cells (see Figure 7B, panel c). Non-labeled fragment ion signals derive from coionized impurities.
